# Specific dilation pattern in placental circulation and the NO/sGC role in preeclampsia placental vessels

**DOI:** 10.3389/fendo.2023.1182636

**Published:** 2023-05-24

**Authors:** Jiaqi Tang, Yumeng Zhang, Ze Zhang, Jianying Tao, Jue Wu, Qiutong Zheng, Ting Xu, Na Li, Zhice Xu

**Affiliations:** ^1^ Institute for Fetology, First Affiliated Hospital of Soochow University, Jiangsu, China; ^2^ Maternal and Child Health Care Hospital of Wuxi, Jiangsu, China; ^3^ Suzhou Municipal Hospital, the Affiliated Suzhou Hospital of Nanjing Medical University, Jiangsu, China; ^4^ Obstetrics and Gynecology, Dushu Lake Hospital Affiliated to Soochow University, Jiangsu, China

**Keywords:** feto-placental circulation, placental vessel, dilation, nitric oxide, preeclampsia, soluble guanylate cyclase (sGC)

## Abstract

**Objective:**

Endothelial functions in controlling blood flow in placental circulation are still unclear. The present study compares vascular dilations between placental circulation and other vessels, as well as between normal and preeclampsia placental vessels.

**Methods:**

Placental, umbilical, and other vessels (cerebral and mesenteric arteries) were collected from humans, sheep, and rats. Vasodilation was tested by JZ101 and DMT. Q-PCR, Western blot, and Elisa were used for molecular experiments.

**Results:**

Endothelium-dependent/derived vasodilators, including acetylcholine, bradykinin, prostacyclin, and histamine, mediated no or minimal dilation in placental circulation, which was different from that in other vessels in sheep and rats. There were lower mRNA expressions of muscarinic receptors, histamine receptors, bradykinin receptor 2, endothelial nitric oxide synthesis (eNOS), and less nitric oxide (NO) in human umbilical vessels when compared with placental vessels. Exogenous NO donors (sodium nitroprusside, SNP) and soluble guanylate cyclase (sGC) activators (Bay41-2272) decreased the baseline of vessel tone in placental circulation in humans, sheep, and rats, but not in other arteries. The sGC inhibitor ODQ suppressed the reduced baseline caused by the SNP. The decreased baseline by SNP or Bay41-2272 was higher in placental vessels than in umbilical vessels, suggesting that the role of NO/sGC is more important in the placenta. NO concentrations in preeclampsia placental vessels were lower than those in control, while no significant change was found in umbilical plasma between the two groups. eNOS expression was similar between normal and preeclampsia placental vessels, but phosphorylated eNOS levels were significantly lower in preeclampsia. Following serotonin, SNP or Bay41-2272-mediated dilations were weaker in preeclampsia placental vessels. The decreased amplitude of SNP- or Bay41-2272 at baseline was smaller in preeclampsia. The decreased amplitudes of ODQ + SNP were comparable between the two groups. Despite higher beta sGC expression, sGC activity in the preeclampsia placenta was lower.

**Conclusion:**

This study demonstrated that receptor-mediated endothelium-dependent dilation in placental circulation was significantly weaker than other vessels in various species. The results, showed firstly, that exogenous NO played a role in regulating the baseline tone of placental circulation *via* sGC. Lower NO production and decreased NO/sGC could be one of the reasons for preeclampsia. The findings contribute to understanding specific features of placental circulation and provide information about preeclampsia in placental vessels.

## Introduction

The fetus is reliant on placental perfusion for effective oxygenation and nutrient supply. Effective interplay of feto-placental circulation is vital to fetal development. The feto-placental circulation is composed of umbilical vessels and the placental vascular tree. Because placental vessels and umbilical vessels have no nervous innervation (1), local autocrine/paracrine factors, such as prostanoids, histamine, and nitric oxide (NO), are of paramount importance for controlling blood flow in the feto-placental circulation (2, 3). The effective regulation of feto-placental vessel tone is important for placental perfusion and fetal development. In human placental–umbilical vessels, several studies suggested that the classical endothelium-related vasodilators, acetylcholine, adenosine diphosphate, insulin, and histamine, caused dose-dependent relaxation (4–9). However, more studies noted that in umbilical and placental vessels from humans and sheep, dilating responses to acetylcholine, bradykinin, and prostacyclin (PGI2) were minimal *in vivo* and *in vitro* (6, 9–15). Thus, the present study used various species, including humans, sheep, and rats, and employed multiple major endothelium-dependent/derived vasodilators (acetylcholine, bradykinin, PGI2, and histamine) to determine the roles of endothelium-related vasodilators in feto-placental circulation.

The baseline tone of umbilical and placental vessels is closely associated with vascular resistance. The enzyme eNOS might play a role in regulating baseline vascular tone in human and sheep umbilical-placental circulation *in vivo* (8, 16). Previous studies demonstrated that N-omega-nitro-L-arginine increased umbilical and placental vascular resistance and arterial pressures, decreased umbilical blood flow *in vivo* (16), and reduced classical endothelium-dependent vasodilation (8), suggesting basal endothelium-related vasodilator plays a role in baseline vessel tension in umbilical–placental circulation. Our previous study clarified that exogenous NO (SNP) regulated the baseline of vessel tone in human umbilical and placental vessels (11). SNP-induced relaxation in umbilical and placental vessels is primarily mediated *via* sGC/cyclic GMP (15, 17). However, the mechanism for the decreased baseline of umbilical and placental vessel tone by SNP is still lacking, which was paid attention to in the present study.

Preeclampsia is a common complication during pregnancy, threatening the health of pregnant mothers and fetuses. The diagnostic guideline for the definition of preeclampsia is according to a document by the Society of Obstetricians and Gynaecologists of Canada (SOGC) (18). The placenta is central to the pathogenesis of preeclampsia (19). Studies have revealed the altered feto-placental circulation in preeclampsia (20). NO is the most prominent vasodilator produced during pregnancy and plays a critical role in vasculogenesis and angiogenesis (21). Some clinical studies found that the serum NO metabolite concentration was reduced in preeclampsia patients (22); however, others reported that it was increased (23). Placental NO is critical for maintaining perfusion in the maternal–fetal–placental circulation during normal pregnancy (24). Previous studies also reported that NO metabolites were changed in preeclampsia placental tissue (24), however the information was still limited to placental vessels. Reduced NO production during pregnancy results in impoverished placental vascularization (25) and placental insufficiency (26). Preeclampsia is closely associated with an imbalance in the L-arginine/NO signaling pathway (4) and altered dilation due to SNPs in placental vessels. However, the role of NO signaling in regulating placental vessel tone is still limited. This present study also focused on NO production and the roles of NO signaling in regulating placental vessel tone in preeclampsia. The data gained would help understand the physiological features of placental vessels and provide important information on preeclampsia onset.

## Methods

### Samples and ethics

#### Humans

The whole or part of the human placenta, umbilical cord, and umbilical blood were obtained from local hospitals (Suzhou Municipal Hospital and First Hospital of Soochow University) following a caesarean section or spontaneous vaginal delivery. Umbilical blood vessels and third-order placental vessels were immediately collected in less than 2 h after being received. Control placental blood vessels (CPV) and umbilical veins (CUV) were collected from normal pregnant women (N = 27) with normal blood pressure. Preeclampsia placental vessels (PEPV) and umbilical veins (PEUV) were collected from a pregnancy with new-onset hypertension and abnormal urine protein (N = 26). The characteristics of human samples are shown in **Table 1**.

**Table 1:** Characteristics of human samples.

	Ctrl (N = 27)	Preeclampsia (N = 26)
Age (year)	30.00 ± 3.79	30.62 ± 5.26
Gestational weeks	39.28 ± 0.82	34.70 ± 3.42*
SBP (mmHg)	118.40 ± 7.60	155.90 ± 10.77*
DBP (mmHg)	71.52 ± 6.00	101.10 ± 8.09*
Proteinuria	–	2+–3+*
Neonatal body weight (g)	3,378 ± 383	2,175 ± 862*
Placenta weight (g)	662 ± 184	519 ± 132*

SBP, systolic blood pressure; DBP, diastolic blood pressure. *P< 0.05.

#### Sheep

Pregnant sheep (Hu sheep, from Zhejiang Huzhou Company, N = 12) at gestational day 122 ± 3 (term: approximately 145 days) were anesthetized by intramuscular injection of atropine (1.5 mg per sheep, intramuscularly) and ketamine (15 mg/kg, intramuscularly). A caesarean operation was conducted under operative anesthesia maintained by spontaneous inhalation of 1.5%–2.0% isoflurane in O_2_. Sheep umbilical veins (SUV), placental vessels (SPV), fetal mesenteric arteries (SMA), and fetal middle cerebral arteries (SMCA) were collected.

#### Rat

Pregnant Sprague–Dawley rats, 5-month-old, (Joinn Suzhou, N = 10) were anesthetized by pentobarbital (50 mg/kg, intraperitoneally). Caesarean delivery was conducted at gestational day 21, and the umbilical veins (Rat-UV) were obtained immediately. Mesenteric arteries (Rat-MA) were collected from maternal rats.

All procedures were approved by The Institute Ethics Committee of First Hospital of Soochow University (No. 278-2020).

### Measurement of vessel tone

Vascular tension of large vessels (human umbilical veins, lumen diameter: about 1,000 μm, and sheep umbilical veins, lumen diameter: about 600 μm) was measured with the JZ101 isometric force transducer (Xinhangxingye Technology, Beijing, China) using Krebs’ solution containing (mmol/L): NaCl 119, KCl 4.7, NaHCO_3_ 25, KH_2_PO_4_ 1.2, CaCl_2_ 2.5, MgSO_4_ 1.0, and D-glucose 11, maintained at 37 °C at pH 7.4 with constant bubbling with 95% O_2_/5% CO_2_. Following 60–120 min of equilibration period, each ring was contracted by KCl (120 mmol/L) three times to test vessel functions and induce maximum constriction. Following the serotonin (5-HT, 10^−4^mol/L)-mediated contractile platform, accumulative acetylcholine (ACh, 10^−9^–10^−4^mol/L), PGI2 (10^−9^–10^−5^mol/L), bradykinin (10^−9^–10^−5^mol/L), histamine (10^−9^–10^−4^mol/L), sodium nitroprusside (SNP, 10^−9^–10^−4^mol/L), or Bay41-2272 (10^−9^–10^−4^mol/L) were added to determine vascular dilation functions, respectively. Dilation responses were normalized by 5-HT-mediated contraction. The regulation of baseline vessel tone was tested using SNP (10^−4^mol/L) with or without ODQ (sGC inhibitor, 10^−5^mol/L), or Bay 41-2272 (sGC activator, 10^−4^mol/L) at steady baseline. Dilation at the baseline of vessel tone induced by the drugs was normalized by the contraction elicited by KCl alone.

Small blood vessel segments were mounted in the myograph system for isometric tension (DMT). The diameter was about 200 μm for human placental vessels, about 200 μm for sheep placental vessels, 120 μm for fetal sheep cerebral arteries, 150 μm for rat mesenteric arteries, 100 μm for rat cerebral arteries, and 250 μm for rat umbilical veins. The segments were immersed in HEPES-solution containing (mmol/L): NaCl 141.85, KCl 4.7, MgSO_4_ 1.7, EDTA 0.51, CaCl_2_·2H_2_O 2.79, KH_2_PO_4_ 1.17, glucose 5.0, and HEPES 10.0; pH 7.4; and gassed with 5% CO_2_ and 95% O_2_. Vessels were given an initial optimum tension by being stretched progressively and adjusted at this level for 60–120 min. The contractile capacity was determined by exposure to a potassium-rich (120 mmol/L) buffer solution. Concentration–response curves to ACh, histamine, PGI2, bradykinin, or SNP were determined following 5-HT. SNP in the presence or absence of ODQ as well as Bay 41-2272 were added to determine the role of NO/sGC in the regulation of basal tension. The rings were used only once for any drug. For all measurements of vessel tone, the next concentration was sequentially added after the platform was achieved. It was usually 3–5 min for each concentration of cumulative doses of drugs.

The optimal tension was set according to the Frank–Starling mechanism. Although resting tension values vary among different vessels, the tension in each vessel was normalized to the KCl-induced maximum contraction or 5-HT-mediated contraction platform. These could minimize the influence of resting tension on different vessels. After measuring vessel tone, HE staining was used to detect the endothelial layer.

### Q-PCR

Total RNA was extracted from tissue samples using Trizol reagents (Takara, Japan). The purity and integrity of the RNA were determined with Nanodrop and agarose gel electrophoresis. RNA (A260/280: 1.8–2.0, 2 μg) was reversely transcribed into cDNA using a first-strand cDNA synthesis kit (Takara, Cat#6210A). All gene primer sequences are shown in **Table 2**. Real-time PCR was performed with SYBR and analyzed on QuantStudio (Thermo Fisher Scientifics) in a 96-well plate. The 2^−ΔΔCt^ method was used to comparatively quantify the mRNA levels of target genes, and *ACTB* was used as an endogenous reference gene.

**Table 2:** Primer sequences.

Primers	Forward	Reverse
*eNOS*	GAGATCCACCTCACTGTAGCTGT	CTCAATGTCATGCAGCCG
*ACTB*	CATGGAGTCCTGTGGCATCCA	CAGGAGGAGCAATGATCTTG
*CHRM1*	CTCTATACCACGTACCTGCTCA	CCGAGTCACGGAGAAGTAGC
*CHRM2*	AACTCCTCTAACAATAGCCTGGC	GTTCCCGATAATGGTCACCAAA
*CHRM3*	CACCATCCTCAACTCCACCA	CAGCTTGTCGGCTTTCCTCT
*CHRM4*	GTTTGTGGTGGGTAAGCGGA	TGCTTCATTAGTGGGCTCTTG
*CHRM5*	CAGGATGGAAGGGGATTCTTAC	AATGGTGATGACTTCCCACAAC
*H1R*	ACAAGATGTGTGAGGGCAACAAG	GTACGGCATACAGCACCAGC
*H2R*	CATCACCGTGGTCCTTGC	AGCTGGTAGATGGCAGAGAAGG
*BDKR1*	CAACTACAGTTGTGAACGCCTTC	GAGCTGGCTCTGGTTGGA
*BDKR2*	CGCCACTCCAGCTCTGG	CATTGAGCATGTCGGCG

### Western blot

Samples were homogenized in liquid nitrogen and thawed in RIPA buffer containing protease inhibitors. The protein abundances of GUCY1A3 (guanylate cyclase 1 soluble subunit alpha), GUCY1B3 (guanylate cyclase 1 soluble subunit beta), and PRKG1 (protein kinase cGMP-dependent 1) were normalized to GAPDH. The primary antibodies for GUCY1A3 (Proteintech, Cat#12605-1-AP), GUCY1B3 (Proteintech, Cat#19011-1-AP), and PRKG1 (Proteintech, Cat#21646-1-AP) were incubated overnight at 4°C. Secondary antibodies (goat anti-mouse for GAPDH, GAM0072; goat anti-rabbit for the others, GAR0072, Multisciences) were incubated for 1 h at room temperature. The immunoreactive bands were visualized using the UVP imaging system. Imaging signals were digitized and analyzed, and the ratio of band intensity to GAPDH was subsequently obtained to quantify relative protein expression.

### ELISA

A commercial enzyme-linked immunosorbent assay kit (Beyotime, Cat#S0021) was used to quantify concentrations of nitric oxide (NO) in human placental vessels, umbilical veins, and umbilical plasma. Due to fact that 1 L of blood plasma is approximately equal to 1,000 g, the comparison of the levels of NO in plasma and tissues was normalized to the concentration per mg of sample. The cGMP expression and sGC activity in human tissues were determined by Beijing Huaying with commercial kits. The color changes were measured with a microplate reader (TECAN), and O.D. values were recorded. The concentration was then determined by comparing the O.D. values of samples to the standard curve.

### Data analysis

Data were expressed as mean ± S.D. An unpaired t-test or two-way ANOVA analysis followed by a Bonferroni *post hoc* test was used to determine statistical significance (p <0.05). The image data were performed with GraphPad Prism (version 9).

## Results

### Dilation in human placental vessels and umbilical vessels

**Figure 1:**
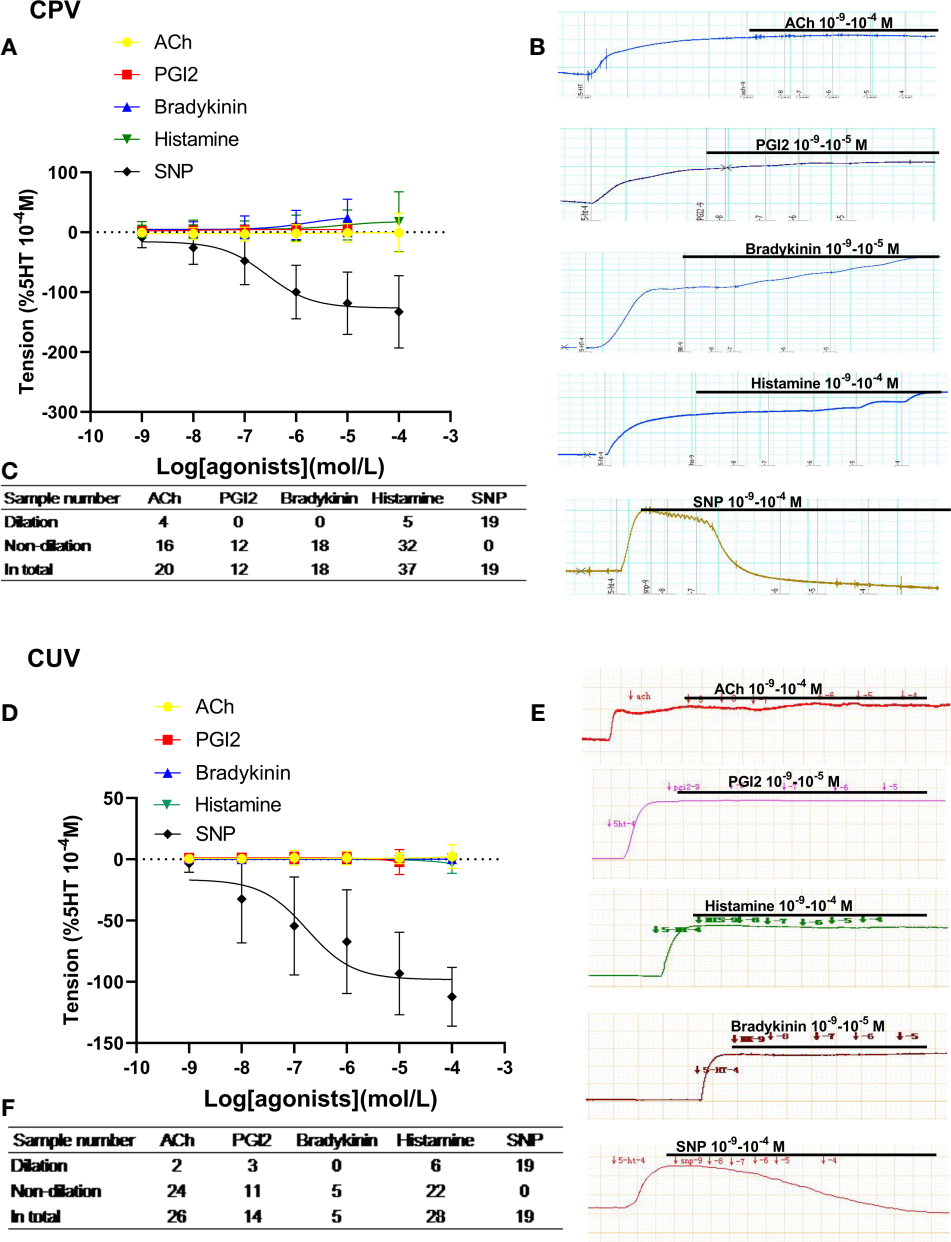
Dilation in normal human placental and umbilical vessels. The doses of vasodilator agents (acetylcholine (ACh), PGI2, bradykinin, histamine, and SNP) mediated dilation responses in normal human placental vessels (CPVs) **(A)** and umbilical veins (CUV) **(D)**. The real-time recording images were also shown **(B, E)**. The tables show the sample numbers used in the experiments, which were the numbers of pregnant women **(C, F)**.

On the vasoconstriction platform induced by 5-HT (10^−4^M), accumulative doses of ACh, PGI2, bradykinin, and histamine mediated none or very weak dilation in normal human placental arteries, which was significantly different from that by SNP (**Figures 1A, B**), indicating very weak endothelial dilation in human placental arteries. A similar phenomenon was also observed in normal human umbilical veins (**Figures 1D, E**). The tables show the sample numbers used in the experiments, which were the numbers of pregnant women (**Figures 1C, F**). These data indicate that classic drug-mediated endothelium-dependent dilation is not a major mechanism in regulating vessel tone in human placental and umbilical vessels.

### Dilation in placental vessels, umbilical vessels, and fetal middle cerebral arteries from sheep

**Figure 2:**
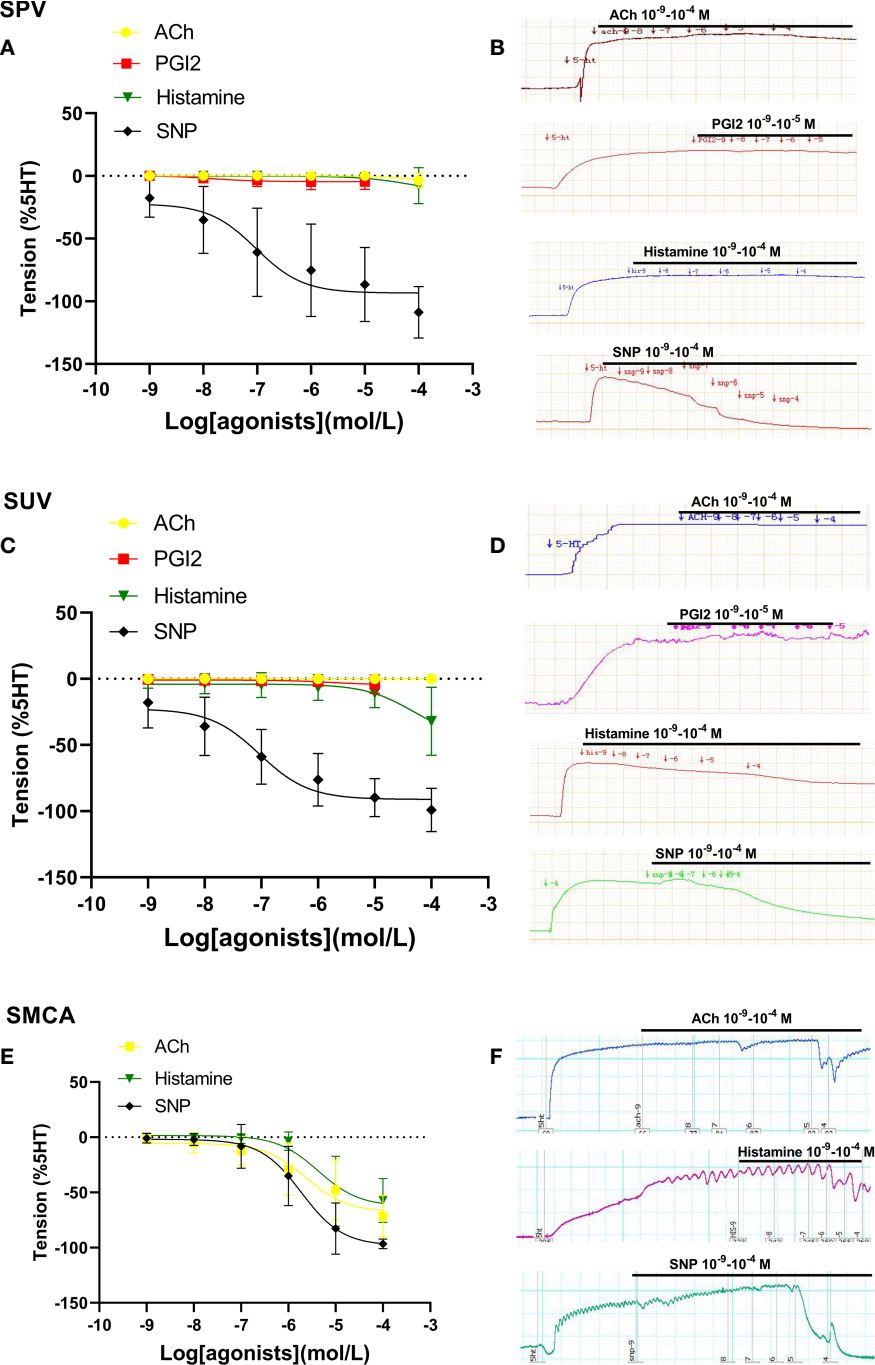
Dilation in sheep placental vessels, umbilical vessels, and the fetal middle cerebral artery. The doses of vasodilator agents (acetylcholine (ACh), PGI2, histamine, and SNP) mediated dilation responses in sheep placental vessels (SPVs) **(A)**, sheep umbilical veins (SUV) **(C)**, and fetal sheep middle cerebral artery (SMCA) **(E)** from sheep species. The real-time recording images were also shown **(B, D, F)**.

**Figure 2** shows vascular responses in sheep. The dilation responses to ACh, PGI2, and histamine in sheep’s placental arteries and umbilical veins were also either very limited or none (**Figures 2A–D**). SNP also showed its obvious vasodilatation in both placental and umbilical vessels in sheep (**Figures 2A–D**). However, in sheep with middle cerebral arteries (non-umbilical or placental vessels), ACh and histamine induced stronger vasodilation (**Figures 2E, F**). These further demonstrated that the endothelial dilation in placental vessels was very weak, which was significantly different from that of non-umbilical or placental vessels.

### Dilation in umbilical veins and mesenteric arteries from rats

**Figure 3:**
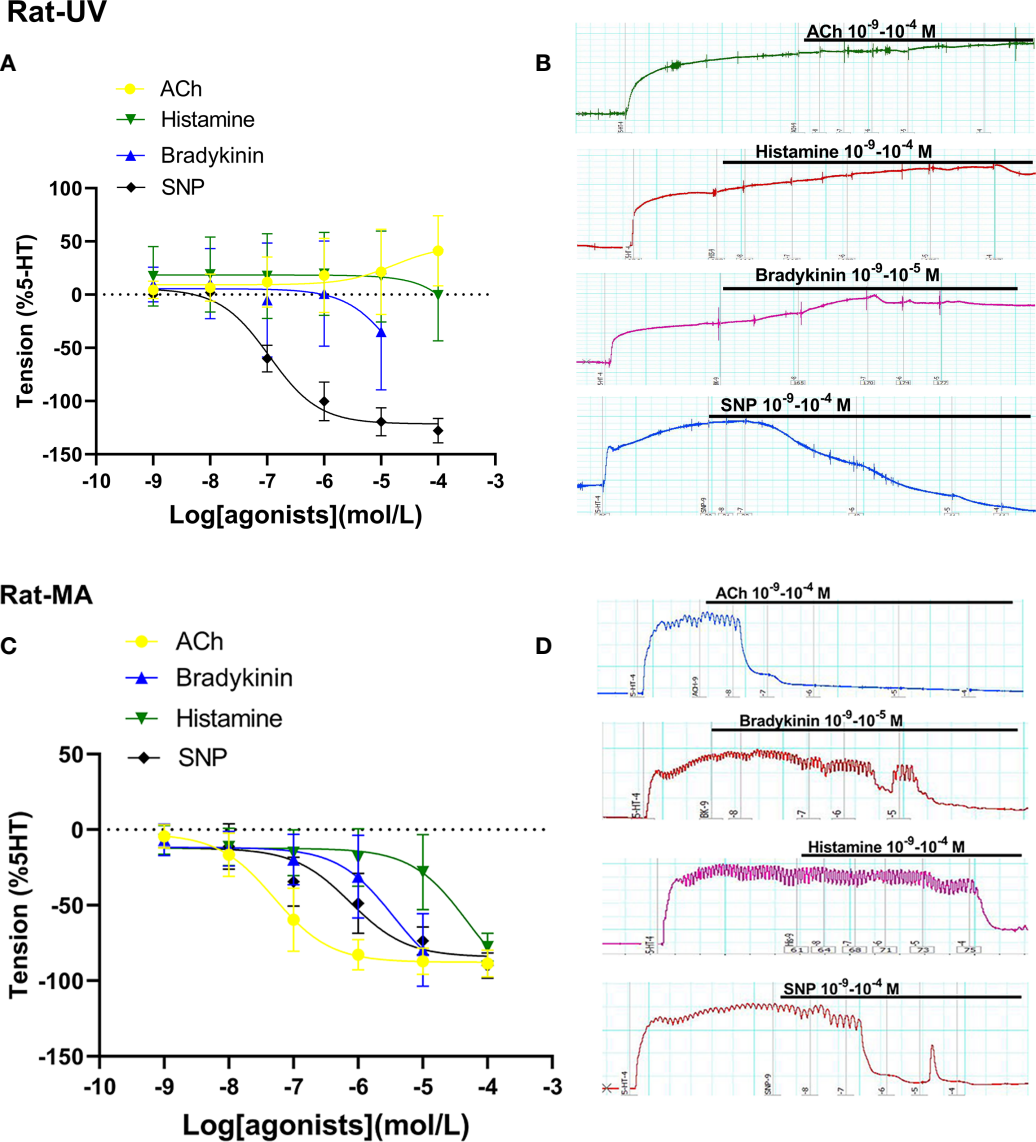
Dilation in umbilical vessels and mesenteric arteries from a rat. Doses of vasodilator agents (acetylcholine (ACh), PGI2, bradykinin, histamine, and SNP) mediated dilation responses in rat umbilical veins (Rat-UV) **(A)** and rat mesenteric arteries (Rat-MA) **(C)**. The real-time recording images were also shown **(B, D)**.

When compared with SNP-caused dilation in rodent species, ACh, histamine, and bradykinin mediated weak dilation in Rat-UV (**Figures 3A, B**) on the constriction platform, while these drugs induced significant vessel dilations in Rat-MA (**Figures 3C, D**).

From human, sheep, and rat species, we could conclude that there is weak endothelium-dependent dilation in umbilical and placental vessels.

### Comparison of molecule expressions between CPV and CUV

**Figure 4:**
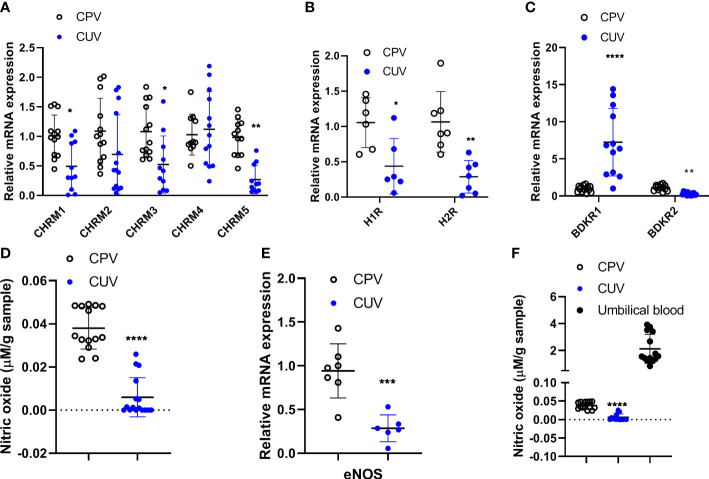
Comparison of molecule expressions between CPV and CUV. Figure shows the mRNA expressions of *CHRM1-5* **(A)**, *H1/2R* **(B)**, *BDKR1/2* **(C)**, and *eNOS* **(E)**, and the concentration of NO in CPV, CUV, and umbilical plasma from normal pregnancy **(D, F)**. *P <0.05;**P <0.01; ***P <0.001; ****P <0.0001.

The mRNA expressions of muscarinic receptors 1/3/5 (*CHRM1/3/5*) (**Figure 4A**), histamine receptors 1/2 (*H1/2R*) (**Figure 4B**), and bradykinin receptors 2 (*BDKR2*) (**Figure 4C**) were significantly lower in umbilical vessels than those of placental vessels (**Figure 4A**), although *CHRM2/4* were the same between the two groups and *BDKR1* was higher in CUV. The concentration of nitric oxide (μmol/g) was lower in CUV (0.006 ± 0.009) than that in CPV (0.038 ± 0.010) (**Figure 4D**). The mRNA expressions of *eNOS* were lower in CUV than in CPV (**Figure 4E**). The NO concentration was significantly higher in umbilical plasma (2.124 ± 1.086 μmol/g) than that of CUV or CPV (**Figure 4F**). These data indicated that the maintenance of vascular tension in the umbilical–placental circulation heavily relies on plasma NO.

### Role of NO/sGC in regulating baseline tone of placental vessels and non-placental vessels

**Figure 5:**
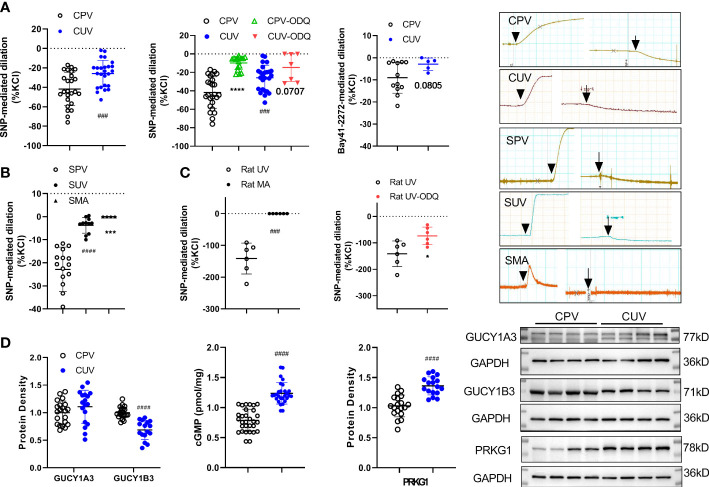
Role of NO/sGC in regulating the baseline tone of placental and non-placental vessels. On the baseline of vessel tone, SNP mediated dilation in several species (**A**—human: normal placental vessels (CPV, n = 25) and umbilical vessels (CUV, n = 25); **B**—sheep: placental vessels (SPV, n = 16), umbilical vessels (SUV, n = 11), and fetal sheep mesenteric arteries (SMA, n = 4), **C**–rat: umbilical vessels and mesenteric arteries) with or without ODQ. Bay 41-2272 induced dilation in CPV (n = 12) and CUV (n = 5) **(A)**. The protein expressions of GUCY1A3, GUCY1B3, cGMP, and PRKG1 in placental vessels and umbilical vessels were shown **(D)**. The real-time recording images were also shown. *P <0.05; ***P <0.001; ****P <0.0001; ^###^P <0.001; ^####^P <0.0001.

SNP (10^−4^M) significantly decreased the vascular baseline tone of normal human placental vessels (CPV) and umbilical veins (CUV) (**Figure 5A**). The decreased amplitude by SNP (normalized to KCl-mediated contraction, %) was higher in CPV (−41.79 ± 17.05) than that in CUV (−25.84 ± 13.65). Analogous to that in humans, the decrease in the baseline tension by SNP was higher in SPV (−22.86 ± 9.65) than that in SUV (−3.74 ± 3.41), while no changes in the baseline tension by SNP were observed in SMA (**Figure 5B**). SNP also decreased the baseline tone in rat umbilical veins (−141.3 ± 48.62), not in the mesenteric arteries of rats (**Figure 5C**). The sGC inhibitor (ODQ) blocked the decreased baseline vessel tone by SNP in CPV (−9.72 ± 7.30), CUV (−14.65 ± 15.13), and rat-UV (−73.41 ± 33.35) (**Figures 5A, C**). And the activator of sGC (Bay 41-2272) also decreased the baseline vessel tone in CPV (−16.5 ± 6.22) and CUV (−2.89 ± 2.80) (**Figure 5A**), and the decrease level was lower in CUV. There was decreased GUCY1B3 protein expression, increased protein expressions of cGMP and PRKG1, and no significant change in GUCY1A3 expression (**Figure 5D**). These indicate that exogenous NO and sGC participate in the regulation of the baseline tension in umbilical and placental vessels. These results indicate there might be non-endothelial dependent NO-sGC pathways in placental vessels that play more important roles in regulating baseline vessel tone in placental vessels.

### Dilation in placental vessels from normal and preeclampsia pregnancy and relative expressions of the molecules

**Figure 6:**
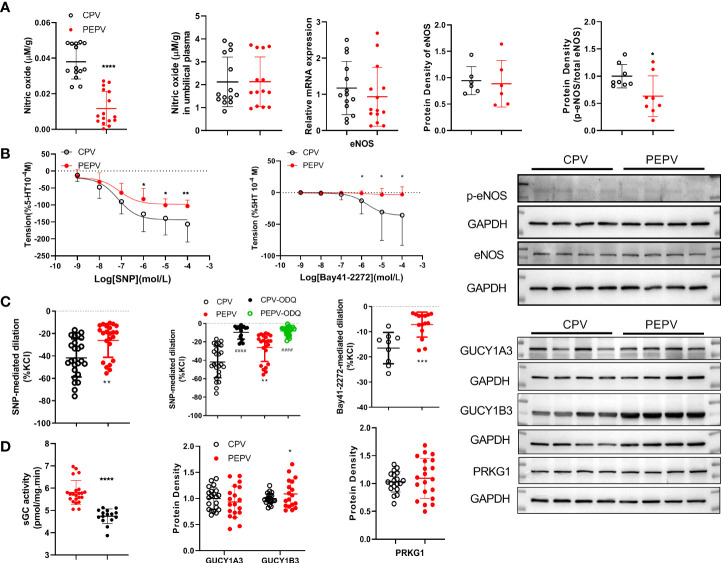
Dilation in placental vessels in normal and preeclamptic pregnancy and related mechanisms. The concentrations of NO in placental vessels and umbilical plasma from both groups were shown **(A)**. The mRNA and protein expressions of eNOS as well as the phosphorylated eNOS are shown **(A)**. On the 5HT-induced vasoconstriction platform, concentration-dependent SNPs, or Bay 41-2272 induced dilation responses in placental vessels from both groups **(B)**. On the baseline of vessel tone, SNP caused dilation in the presence or absence of ODQ **(C)**. Bay 41-2272 decreased the baseline of placental vessel tone **(C)**. The sGC activity and protein expressions of GUCY1A3, GUCY1B3, and PRKG1 were shown **(D)**. *P <0.05; **P <0.01; ***P <0.001; ****P <0.0001; ^####^P <0.0001.

**Figure 6A** shows that the concentrations of NO in PEPV were lower than those in CPV. No significant differences were found in nitric oxide concentrations in umbilical plasma from both groups. There was a lower eNOS mRNA expression in PEPV while no significant differences in eNOS protein expression. The ratio of phosphorylated eNOS protein expression to total eNOS was decreased in PEPV. These demonstrated the decreased eNOS-NO production in preeclampsia placental vessels.

**Figure 6B** presents that following 5-HT-induced constriction, SNP or Bay 41-2272-mediated dilation responses were weaker in preeclampsia placental vessels (PEPV) than in normal placental vessels (CPV). **Figure 6C** shows that SNP- or Bay 41-2272-decreased amplitude on the baseline vessel tone was less in PEPV than that of the CPV. The sGC inhibitor (ODQ) significantly blocked SNP-mediated reduction on the baseline tension in CPV and PEPV. Although there was no significant difference in GUCY1A3 and PRKG1 protein expressions as well as higher GUCY1B3 protein expression, the sGC activity in preeclampsia placenta vessels was obviously declined (**Figure 6D**).

## Discussion

The present study revealed that almost all major endothelial-related vasodilators mediated weak relaxation in human umbilical veins and placental vessels when compared to non-placental vessels. The only healthy human non-placental vessels available were umbilical cord vessels, and to fully illustrate the specificity of placental vessels, animal mesenteric arteries and cerebral arteries were used as non-placental vessels for comparison. Umbilical veins were used because they were important for supplying oxygen and nutrients to fetuses. The placental vessels, umbilical veins, and other arteries were easy to collect from sheep and rats in our lab, which further demonstrated that endothelium-related vascular dilation in umbilical and placental circulation was limited. Analogous to previous studies (6, 10–12, 27–29), the present study was among the first group of studies to demonstrate minimal endothelial dilation in umbilical and placental vessels by using various species and multiple vasodilators. The results not only contribute to further understanding the special vascular physiological patterns of feto-placental circulation but also increase our understanding of the mechanisms of diseases in pregnancy such as preeclampsia.

When comparing umbilical vessels, there was higher NO production in placental vessel tissue. Others’ and our previous studies have demonstrated different vascular functional patterns between the placental and non-placental vascular systems (10, 11). For example, ACh has been shown to have a special constriction behavior in human umbilical vessels (12), without any significant effect on placental vessels (30), since this drug is a typical classic vasodilatation agent for other organs such as the MA. The special functional features of placental vessels suggest a need for maintaining a relatively weaker endothelial-dependent NO system in placental circulation.

Paulick and colleagues proposed that the inability of the endothelial-dependent vasodilators in the umbilical–placental circulation could be due to a lack of relative receptors in the umbilical and placental vasculature (15). The present study added novel evidence that the mRNA expressions of relative G protein-coupled receptors (including muscarinic receptors, histamine receptors, and bradykinin receptors) were expressed in human umbilical veins and placental vessels and firstly revealed that CHRM1/3/5, H1/2R, and BDKR2 mRNA expressions were higher in placental vessels than umbilical vessels. In addition, there was higher eNOS mRNA and protein expression in placental vessels. Based on the nitrite (product of NO metabolism) levels, the present study speculated that placental vessel tissue could produce relatively higher NO accompanied by a weaker endothelial-dependent NO system, suggesting that non-endothelial NO may play a role in placental circulation.

Our previous study demonstrated that the NO donor, SNP, could decrease the baseline tone of placental circulation but could not affect the baseline vessel tones in non-placental vessels such as mesenteric arteries (11), exhibiting that umbilical and placental vessels can respond to NO despite its endothelial-dependent NO effects being weaker and demonstrating that vascular baseline tension in the placenta is regulated by non-endothelial NO, which is significantly different from non-placental blood vessels. These suggested that placental tissues, instead of placental endothelial cells, could produce more NO, which played a role in regulating placental vessel tone. SNP-induced relaxation in human umbilical vessels was suggested to be primarily mediated *via* the sGC/cGMP pathway (17). The present study was the first to show that the regulation of baseline tone by SNP was also mediated *via* sGC in placental circulation, answering the question “how SNP as a NO donor produces a big dent (reduced baseline tension) in umbilical and placental blood vessels.”

In contrast to previous studies on non-placental vessels, the role of NO/sGC signaling was determined in placental vessels. The reduced amplitude was significantly higher in placental vessels, illustrating a relatively stronger endothelial-independent functional NO/sGC pathway in regulating the baseline tone of placental vessels, which was mainly *via* a higher GUCY1B3 protein. It is well known that the low impedance of placental circulation is important in maintaining blood perfusion during normal pregnancy (2). Nitric oxide inhibitors N omega-nitro-L-arginine markedly potentiated the constrictor effects and increased fetal vascular basal perfusion pressure (31). Our findings further clarified that the NO/sGC pathway played a vital role in regulating the baseline vessel tone in placental vessels. Interestingly, higher concentrations of NO metabolites and stronger responses of the NO/sGC pathway in regulating the baseline vessel tone in the placenta were noted. Thus, it was suggested that these might be due to physiological adaptations in the placenta for maintaining low impedance and adequate perfusion.

The nitrite concentration in umbilical plasma was about 2.124 μmol/g (approximately 2 μmol/L), which was similar to some studies (32, 33). The biochemical experiment in the present study first compared nitrite concentrations (a product of NO metabolism) between the placental circulation and placental vessels and found that umbilical plasma nitrite (a product of NO metabolism) levels were higher than those in placental vessels. The umbilical blood flow is influenced by the fetal heart, which beats about 140 times per minute. The placenta is next to the umbilical cord. In fact, umbilical blood vessels enter the placenta. With the rapid fetal heartbeat, the umbilical blood is quickly and continuously flowing to the placenta. Thus, NO in the umbilical blood can reach the placenta quickly. In addition, the placenta is bathed in maternal blood sinuses. Considering NO is a material that can easily diffuse into placental vessels, there may be possibilities that non-endothelial NO in the placenta could be from umbilical or maternal blood that is either next to the placenta or in the placenta. This interesting finding suggests that exogenous NO from blood flow in the umbilical–placental circulation may be a major supply of endothelial-independent NO for the regulation of placental vasodilation, especially for maintaining vascular baseline tension in the placenta.

Pregnancy-induced hypertension has been linked to poor perfusion in the placental circulation (34, 35), which could be associated with NO signaling (36). Placental NO deficiency in preeclampsia patients might contribute to alterations in blood perfusion in the placental circulation (37). The present study compared eNOS/NO production and found that reduced phosphorylated eNOS protein expression decreased endothelium-dependent NO production in placental vessels in preeclampsia. The role of the NO/sGC pathway in regulating vessel tone was weaker in the preeclampsia placental vessels when compared with normal placental vessels, which might potentiate vessel tone and reduce placental perfusion in preeclampsia. On the contraction platform, SNP-mediated dilations in placental vessels were attenuated in preeclampsia placental vessels. In preeclampsia, the effect of the NO/sGC pathway on regulating vascular baseline tone was also reduced, associated with decreased sGC activities (11). These further indicated that the regulation by NO/sGC was insufficient in preeclampsia placental vessels. The increased GUCY1B3 protein expression might be negative feedback to the weaker NO/sGC pathway in preeclampsia. The lower NO production and weaker role of the NO/sGC pathway might contribute to the poor perfusion in the placental vessels of preeclampsia, leading to the onset of hypertension during pregnancy.

One of the limitations of the present study was that it was impossible to collect normal systemic vessels from humans due to ethical concerns, so systemic vessels from animal models were the only choice. The gestation time of PE patients is usually shorter than that of normal controls. The comparisons between the normal control and PE are always made with the inevitable shortcoming of different gestational ages. Non-receptor-mediated endothelium-dependent relaxation and shear stress are also responsible for endothelium-dependent dilation in the body, which is worthy of further investigation. The other limitation is that the animal arteries collected from different species were not age-matched.

In conclusion, the receptor-mediated endothelial-dependent dilation in umbilical–placental circulation was minimal compared to non-umbilical or non-placental vessels. The NO/sGC pathway was more important in regulating baseline tone in placental vessels than that in umbilical vessels. The lower NO production and weaker role of NO/sGC in placental vessels could be one of the causes of preeclampsia onset.

## Data availability statement

The original contributions presented in the study are included in the article/supplementary material. Further inquiries can be directed to the corresponding authors.

## Ethics statement

The studies involving human participants were reviewed and approved by The Institute Ethics Committee of First Hospital of Soochow University. The patients/participants provided their written informed consent to participate in this study. The animal study was reviewed and approved by The Institute Ethics Committee of First Hospital of Soochow University.

## Author contributions

JQT designed the experiments. JQT, YMZ, and ZZ did the measurement of vessel tone. YMZ and ZZ did the molecule tests. JYT and JW collected the samples in clinic. QTZ and TX gave help in raising the rats and sheep. JQT wrote the manuscript. NL and ZCX revised it. All authors listed have made a substantial, direct, and intellectual contribution to the work and approved it for publication.
